# Role of Pulse Oximetry Screening for Term Healthy Newborns During Transitional Period to Detect Critical Congenital Heart Disease (CCHD): A Prospective Observational Study in a Tertiary Care Hospital

**DOI:** 10.7759/cureus.108873

**Published:** 2026-05-15

**Authors:** Hardik Shah, Dhanani Vishakha Mansukhlal, Nidhi Modi, Sushmita Devi Haodijam, Alpesh Singhvi, Jaykumar Rameshbhai Jasani, Naiya Bhavsar

**Affiliations:** 1 Department of Pediatrics, Dr. N.D. Desai Faculty of Medical Science and Research, Dharmsinh Desai University, Nadiad, IND; 2 Department of Pediatrics, Shija Academy of Health Sciences, Imphal, IND; 3 Department of Pediatrics and Neonatology, Surat Diamond Association (SDA) Hospital, Surat, IND; 4 Department of Pediatrics, Jasani Children Hospital, Surat, IND; 5 Department of Respiratory Medicine, Dr. N.D. Desai Faculty of Medical Science and Research, Dharmsinh Desai University, Nadiad, IND

**Keywords:** congenital heart disease, critical congenital heart disease, early screening, newborn, pulse oximetry, screening

## Abstract

Background: Early detection of critical congenital heart disease (CCHD) remains a challenge, especially in settings with early postnatal discharge. Pulse oximetry screening is a simple, non-invasive method that can aid in early diagnosis, but optimal timing in resource-limited settings requires evaluation.

Objective: This study aims to assess the effectiveness of pulse oximetry screening at 15 minutes and six hours of life in identifying persistent hypoxemia and detecting CCHD or other early echocardiographic cardiac findings among term healthy newborns.

Methods: This prospective observational study was conducted over six months in a tertiary care hospital and included 530 term newborns with birth weight ≥2.5 kilograms (kg). Oxygen saturation (peripheral capillary oxygen saturation (SpO₂)) was measured at 15 minutes and six hours of life. Newborns with SpO₂ <95% were reassessed, and those with persistent abnormal values or limb saturation difference ≥3% underwent echocardiography. Data were analyzed using IBM SPSS Statistics for Windows, Version 21 (Released 2012; IBM Corp., Armonk, New York).

Results: Of 530 newborns, 81 (15.3%) had SpO₂ <95% at 15 minutes. At six hours, 75 were normalized, while six newborns (1.13%) had persistent abnormal screening findings and underwent echocardiography. Among these, two newborns (0.37%) were diagnosed with CCHD, and four had patent ductus arteriosus (PDA) detected on early echocardiography. Because echocardiography was performed during the early neonatal transitional period, PDA findings may have been physiological and were not confirmed as persistent CHD by follow-up echocardiography. Newborns delivered by lower segment cesarean section (LSCS) showed a higher incidence of low SpO₂ compared to normal vaginal delivery (NVD).

Conclusion: Early pulse oximetry screening at 15 minutes and six hours is a practical approach for identifying term newborns with persistent hypoxemia who may require further cardiac evaluation before discharge. This approach may be feasible for routine neonatal care, particularly in settings with early discharge practices; however, larger studies with follow-up are needed to define diagnostic accuracy, false-negative rates, and the clinical significance of early PDA findings.

## Introduction

Congenital heart disease (CHD) is a varied collection of structural cardiac defects observed in infancy and is a major cause of disease and mortality among infants across the world. CHD rates have been estimated to be 6-8 per 1000 live births, with a large percentage requiring early diagnosis and intervention to avoid adverse outcomes [1]. Critical congenital heart disease (CCHD) is one of these subgroups, comprising severe defects that require surgical or catheter-based intervention in the first year of life. Late detection of this condition may result in life-threatening complications, such as circulatory collapse, and increased perioperative risk [2].

Despite the availability of prenatal and postnatal clinical examination and advances in prenatal imaging techniques, early diagnosis of CHD remains challenging. Routine physical examination alone lacks sufficient sensitivity, especially in apparently healthy neonates, because many serious malformations are not immediately evident in the early neonatal period [3]. Studies evaluating diagnostic accuracy have shown that reliance on clinical examination alone may result in missed cases, highlighting the need for adjunctive screening tools [4]. Pulse oximetry screening has emerged as an accurate and efficient method for early detection of CCHD. It enables identification of hypoxemia, which may not be clinically apparent, thereby facilitating the timely detection of underlying cardiac defects [5]. Systematic reviews and meta-analyses have demonstrated high specificity with acceptable sensitivity, supporting its role as a valuable addition to existing screening protocols [6]. Additionally, pulse oximetry has utility beyond cardiac screening, aiding in the early detection of other critical neonatal conditions such as sepsis and respiratory disorders [7].

Economic evaluations have indicated that routine screening is a cost-effective strategy to reduce morbidity and mortality associated with undiagnosed CCHD [8]. Large-scale implementation studies have also demonstrated its feasibility across diverse populations, including resource-limited settings where access to advanced diagnostic modalities is restricted [9]. Despite these advantages, challenges remain in optimizing screening protocols, particularly regarding timing. Current recommendations generally suggest screening at or after 24 hours of life to minimize false-positive results [6]. However, in many developing regions, early postnatal discharge limits the feasibility of delayed screening. Newborns are often discharged within 24 hours, increasing the likelihood of missed diagnoses if screening is postponed [10].

The integration of pulse oximetry with other diagnostic modalities has gained attention, particularly its role in enhancing the effectiveness of cardiac auscultation [11]. Advances in diagnostic technologies, including artificial intelligence-based tools, have also shown potential in improving the rapid detection of specific cardiac defects such as patent ductus arteriosus (PDA) [12]. These developments reflect the evolving landscape of neonatal cardiac screening and underscore the need for adaptable approaches tailored to diverse healthcare contexts. Healthcare disparities further influence outcomes in infants with CHD, as differences in access to specialized care, socioeconomic status, and healthcare infrastructure significantly affect diagnosis, management, and survival [13]. Accessible screening methods such as pulse oximetry can help mitigate these disparities by enabling early referral and timely intervention.

Timely management of CCHD is essential and may involve specialized care pathways to stabilize affected infants. Early diagnosis facilitates appropriate planning for interventions such as surgical correction or catheter-based procedures, which are critical for improving survival and reducing long-term complications [14]. Most existing studies have focused on screening performed at or after 24 hours of life, with limited data available on the diagnostic utility of screening within the first few hours after birth [15]. Therefore, evaluating pulse oximetry screening at 15 minutes and six hours of life is necessary to determine its effectiveness in early detection of CHD in term newborns.

Objective of the study

This study aims to assess the efficacy of pulse oximetry screening at 15 minutes and six hours of life for identifying persistent hypoxemia and detecting CCHD or other early echocardiographic cardiac findings in term healthy infants. The study also aims to determine the incidence of confirmed CCHD and screen-detected cardiac findings and evaluate the feasibility of implementing early screening prior to discharge in routine neonatal care settings.

## Materials and methods

Study design

It was a prospective observational hospital-based study conducted in the labor room of KLE's Dr. Prabhakar Kore Hospital and Medical Research Centre, Belagavi, India.

Study population

All healthy newborns who were born during the study were assessed for eligibility. Newborns admitted to the postnatal ward at a gestational age of 37 weeks or older and with a birth weight above 2.5 kg were included following the stabilization period. Premature infants, infants with a birth weight under 2.5 kg, those with life-threatening congenital anomalies, and those whose prenatal diagnosis of CHD were excluded from the study. A total of 530 newborns who met the inclusion criteria were enrolled in the study.

Procedure and data collection study

All enrolled newborns received standard care in accordance with the Neonatal Resuscitation Program immediately after birth. A Planet 55 Larsen and turbo microprocessor-based multichannel handheld pulse oximeter equipped with a neonatal DURA-Y SpO₂ sensor probe was used to perform pulse oximetry screening. Oxygen saturation was measured at 15 minutes and six hours after birth by a trained resident doctor. The readings were taken at the right upper limb and the right lower limb, with the probe on the wrist or palm and on the sole, respectively. Only through the stabilization of the waveform on the monitor were readings taken to ensure the waveform remained stable.

Screening protocol

An SpO₂ value greater than 95% was considered normal. Newborns whose saturation exceeded this level at 15 minutes were not followed up further for pulse oximetry screening. Newborns with SpO₂ values between 90% and 95%, as well as those with SpO₂ values below 90%, were reassessed at six hours of life. At six hours, SpO₂ values greater than 95% were considered normal. Values below 90% were considered abnormal and led to referral for echocardiography. A difference between upper and lower limb saturation was measured when SpO₂ was between 90% and 95%. Any upper-lower limb saturation difference below 3% was considered normal, whereas a difference of ≥3% was considered a persistent abnormality on screening and led to referral for echocardiographic evaluation. Persistent abnormal screening at six hours was therefore defined as either SpO₂ <90% or SpO₂ between 90% and 95% with an upper-lower limb saturation difference of ≥3%. All indicated echocardiography tests were conducted within 24 hours by a pediatric cardiologist.

Outcome measures

The primary outcome was the identification of newborns with persistent abnormal pulse oximetry screening who required echocardiographic evaluation, including the detection of confirmed CCHD and other early echocardiographic cardiac findings. Other observations involved the oxygen saturation patterns at 15 minutes and six hours of life and how these were related to the mode of delivery.

Statistical analysis

The method used to analyze data was the IBM SPSS Statistics for Windows, Version 21 (Released 2012; IBM Corp., Armonk, New York). Nominal variables were represented by percentages. The median and the interquartile range were used to summarize ordinal data. The continuous variables were those of normal distribution, and they were presented as means and standard deviations.

## Results

Baseline characteristics

A total of 530 term newborns with a birth weight of ≥2.5 kg were included in the study. The mean gestational age of the women studied was 38.16 weeks, with a standard deviation of 2.038 weeks. The average weight of the babies at birth was 2.752 with a standard deviation of 0.322. There were 289 male and 241 female newborns enrolled. Table 1 summarizes the demographic characteristics of the study population and the clinical characteristics of the group of participants (baseline).

**Table 1:** Demographic Details SD: Standard deviation, SpO₂: Peripheral capillary oxygen saturation, N: Number, NVD: Normal vaginal delivery, LSCS: Lower segment cesarean section

Variable	Parameter	Values
Gestational Age	Mean (SD)	38.16 wk (2.038)
Birth Weight	Mean (SD)	2.752 (0.322)
Gender	Male (N)	289
	Female (N)	241
SpO_2_ at 15 minutes	<95% mean (SD)	93.46% (1.448)
	>95% mean (SD)	98.24% (1.285)
SpO_2_ at 6 hours	<95% mean (SD)	90.04% (1.312)
	>95% mean (SD)	96.65% (1.665)
Type of Delivery	NVD (N)	266
	LSCS (N)	264

Pulse oximetry screening at 15 minutes and six hours

**Figure 1:**
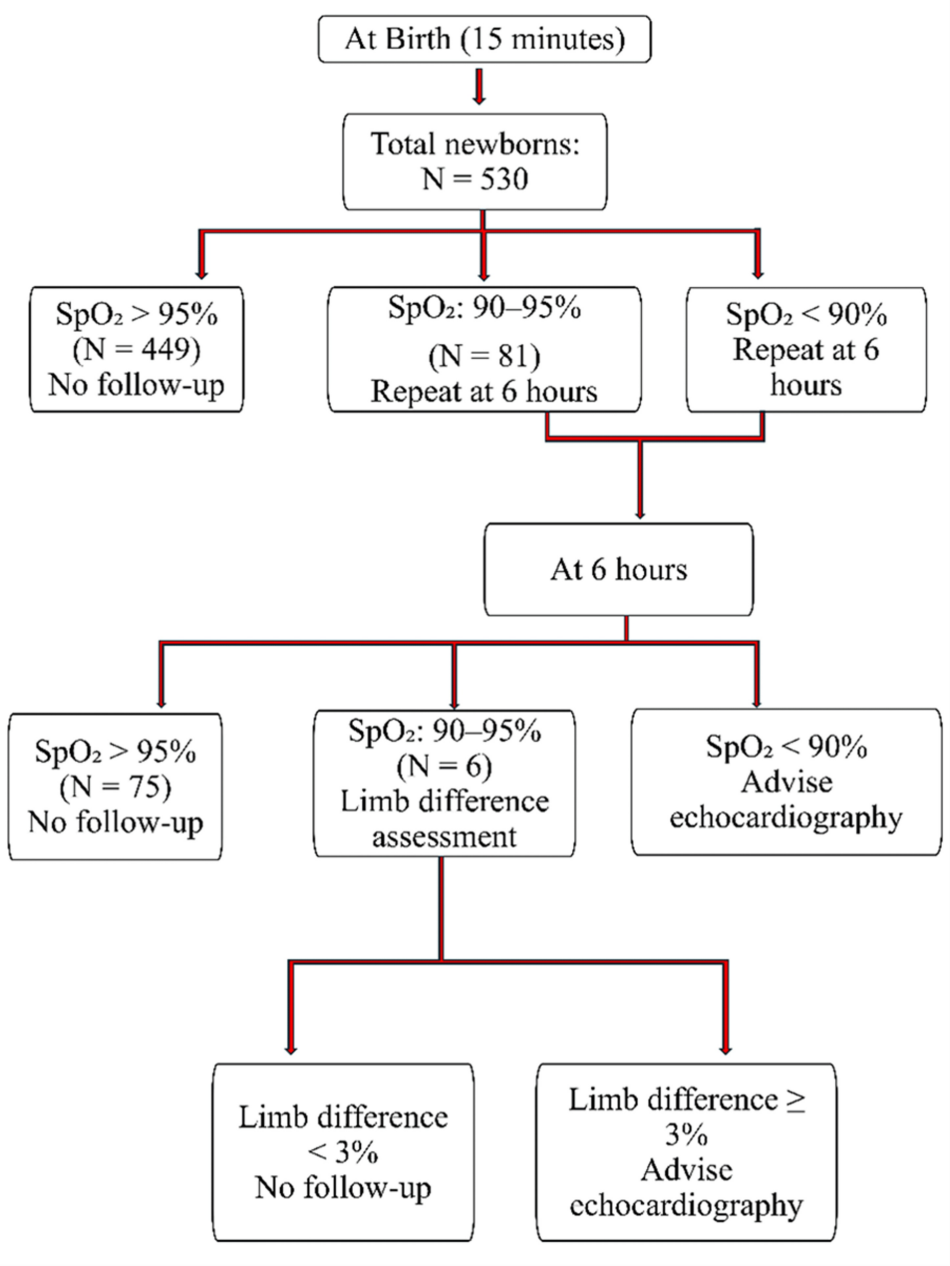
Flowchart Illustrating the Pulse Oximetry Screening Protocol SpO₂: Peripheral capillary oxygen saturation, N: Number of newborns

Of the 530 newborns, 449 (84.7%) had an SpO₂ value >95% at 15 minutes of life and did not require follow-up. The remaining 81 newborns (15.3%) with SpO₂ <95% were reassessed six hours after birth. Of these 81 newborns, 75 (92.6%) improved to an oxygen saturation above 95% and were considered normal at six hours. Figure 1 depicts the pulse oximetry screening protocol and distribution of outcomes.

The remaining six newborns (7.4%) maintained SpO₂ saturation levels below 95% and limb saturation differences of 3% or more and were then referred for further testing with echocardiography. The mean SpO₂ at 15 minutes among newborns with saturation below 95% was 93.46% (SD 1.448), while those with saturation above 95% had a mean of 98.24% (SD 1.285). At six hours, the mean SpO₂ among those with saturation below 95% was 90.04% (SD 1.312), whereas those above 95% had a mean value of 96.65% (SD 1.665). Table 2 shows the distributions of oxygen saturation levels at 15 minutes and six hours of life.

**Table 2:** Measured Saturations at 15 Minutes and Six Hours SpO₂: Peripheral capillary oxygen saturation, N: Number

Parameter	Category	Number (N)
SpO₂ at 15 minutes	<95%	81
	>95%	449
SpO₂ at 6 hours	<95%	6
	>95%	75

Association with the mode of delivery

Out of 530 newborns, 266 were delivered by normal vaginal delivery (NVD) and 264 by lower segment cesarean section (LSCS). At 15 minutes of life, 24 newborns delivered by NVD and 57 delivered by LSCS had SpO₂ values less than 95%. There were two and four newborns, respectively, in the NVD and LSCS groups who had SpO₂ values of less than 95% at six hours of life. In total, 57 (21.6) out of LSCS newborns had low oxygen saturation at 15 minutes of birth compared to 24 (9.02) out of NVD newborns. Newborns delivered by LSCS had a higher frequency of low oxygen saturation during the six hours following delivery compared to those born through NVD. The association between mode of delivery and oxygen saturation levels at different time points is shown in Table 3.

**Table 3:** Types of Delivery and SpO2 Saturation SpO₂: Peripheral capillary oxygen saturation, NVD: Normal vaginal delivery, LSCS: Lower segment cesarean section, N: Number

Types of Delivery	Total Number of Deliveries	SpO₂ at 15 min	Number of Deliveries	SpO₂ at 6 hours	Number of Deliveries
NVD	266	<95%	24	<95%	2
		>95%	242	>95%	22
LSCS	264	<95%	57	<95%	4
		>95%	207	>95%	53

Detection of CHD

Echocardiographic investigation was performed within 24 hours of birth in the six infants who had persistent abnormal screening results at six hours of life, defined as SpO₂ remaining between 90% and 95% with an upper-lower limb saturation difference of ≥3%; no infant in this group had SpO₂ <90% at the six-hour assessment. Echocardiography revealed cardiac findings in all six infants. Out of these, two newborns were diagnosed with CCHD, namely, transposition of the great arteries and tricuspid atresia. The other four newborns had PDA detected on early echocardiography. Because echocardiography was performed during the early neonatal transitional period, these PDA findings may have been physiological rather than persistent pathological PDA. Follow-up echocardiography to confirm ductal closure, persistence, or need for intervention was not included in the study protocol; therefore, PDA should not be interpreted as confirmed persistent CHD in this study. The study population had a total incidence of screen-detected cardiac findings of 1.13%, including a CCHD incidence of 0.37%. If PDA cases are excluded as potentially physiological transitional findings, the incidence of confirmed CCHD was 0.37%. Table 4 provides detailed echocardiographic results for newborns with abnormal screening results.

**Table 4:** Echocardiographic Findings of Newborns With Abnormal Pulse Oximetry Screening SpO₂: Peripheral capillary oxygen saturation, PDA: Patent ductus arteriosus, PFO: Patent foramen ovale, TGA: Transposition of the great arteries, PAH: Pulmonary arterial hypertension

SPO₂ at 15 min	SPO_2_ at 6 hours		ECHO
	Right Upper Limb	Right Lower limb	
88%	94%	90%	PFO with Small Closuring PDA
85%	89%	92%	TGA with Severe PAH
90%	94%	89%	Moderate to large PDA with PFO
85%	93%	88%	Moderate PAH with Tricuspid Atresia
85%	91%	94%	Mild to moderate PDA
92%	94%	90%	Large PDA with Severe PAH

Summary of screening outcome

Pulse oximetry screening at 15 minutes with reassessment at six hours of life allowed identification of newborns with persistent hypoxemia who required further cardiac evaluation. A low percentage of newborns required echocardiography, which identified two cases of CCHD and four early PDA findings. Because PDA was detected during the early neonatal transitional period and follow-up echocardiography was not performed, these PDA findings should be interpreted cautiously. These findings support the usefulness of early screening for identifying newborns who require further cardiac evaluation, rather than proving definitive CHD detection in all abnormal screens.

## Discussion

In the current study, early pulse oximetry screening at 15 minutes and 6 hours of life was evaluated as a practical approach to identify persistent hypoxemia and detect CCHD or other early echocardiographic cardiac findings in term newborns [16]. The results suggest that early screening may help identify newborns requiring further cardiac evaluation before discharge, particularly in settings where early postnatal discharge is common.

Pulse oximetry has been well accepted as a reliable screening method for CCHD because it can be used to detect hypoxemia even prior to the occurrence of clinical symptoms [17]. Past studies have demonstrated that pulse oximetry screening is highly specific and has a fairly good sensitivity, making it applicable to regular neonatal screening programs [18]. In the current study, 15-minute screening identified newborns with low oxygen saturation, while repeat screening at six hours helped distinguish transient transitional hypoxemia from persistent abnormal screening findings that required echocardiographic evaluation. This two-step screening method is consistent with current strategies that underscore the need for repeated measurements to enhance screening interpretation [19]. The prevalence rate of screen-detected cardiac findings in the current trial was 1.13% when both CCHD and early PDA findings were included, while the prevalence of confirmed CCHD was 0.37%. The four PDA cases were detected by echocardiography performed within 24 hours of birth, during the early neonatal transitional period. Because follow-up echocardiography was not included in the study protocol, the persistence of a PDA, spontaneous closure, or the need for intervention could not be assessed.

Therefore, these PDA findings may have represented physiological transitional PDA rather than pathological CHD and should be interpreted cautiously when estimating the true positive rate for CHD. If PDA cases are excluded as potentially physiological findings, the confirmed CCHD detection rate in this cohort was 0.37%. This finding is consistent with existing literature indicating that PDA is a common cardiac condition among neonates, especially during the initial transition period [20]. Although many PDA cases may close spontaneously, timely identification remains useful for ensuring appropriate clinical monitoring and follow-up when indicated.

The study has also shown that a considerable percentage of newborns with low oxygen saturation at 15 minutes recovered by six hours of age, indicating the dynamic physiological changes a baby undergoes during the transitional period after birth. The outcome makes the value of repeat screening to minimize the rates of false-positive screening clear, as was also established in past studies comparing screening protocols [18]. A key finding of this study was the increased percentage of newborns who were found to have low oxygen saturation after LSCS compared with those delivered by NVD. This could be due to the delay in clearance of lung volume and the change in cardiopulmonary adaptation during cesarean births. Other studies have also observed similar associations, implying that the mode of delivery may influence early oxygen saturation levels during the neonatal transitional period and should be taken into consideration when interpreting the screening results [10].

The practicability of the application of early pulse oximetry screening is especially applicable in low- and middle-income environments, where the option of screening at 24 hours of life is diminished by the practice of early discharge. A study has revealed pulse oximetry to be a simple and inexpensive device with application potential in everyday neonatal practice, even in resource-constrained environments [8]. A key strength of the present study is the practical nature of the two-step screening approach, which uses a non-invasive, low-cost, and readily available tool that can be implemented in routine postnatal care without the need for advanced infrastructure. This makes the protocol particularly relevant for settings where early discharge is common and delayed screening at or after 24 hours may not be feasible. The current study supports this approach by showing that screening during the first six hours can identify newborns who may need additional cardiac evaluation before discharge, thereby reducing the risk of missed clinically relevant abnormalities. Recent developments in screening methods have also taken into consideration the integration of pulse oximetry with other modalities like cardiac auscultation to augment the precision of the diagnoses [12,13]. Technologies, such as artificial intelligence-based diagnostic applications, have also demonstrated the possibility of enhancing early disease diagnosis of certain heart-related conditions [15].

Early diagnosis of CCHD is clinically important. Detection can enable timely referral, stabilization, and intervention, which are essential to enhance survival and long-term outcomes [20]. Later diagnosis has been found to result in greater morbidity, such as shock-induced and heart failure, highlighting the need to implement effective screening strategies. Patients having unequal access to special cardiac treatment illustrates the necessity of universally applicable screening tools that could be used to facilitate the early detection and referral [18]. To sum up, pulse oximetry screening at 15 minutes followed by reassessment at six hours appears to be a useful approach for identifying term newborns with persistent hypoxemia who may require further cardiac assessment before discharge. Such a practice may be particularly relevant in settings where early discharge is common and can be considered for integration into routine neonatal care, while larger studies with follow-up are needed to confirm diagnostic accuracy and clinical outcomes.

Limitations and future directions

The current study has its limitations that must be taken into account during the interpretation of the results. The study was limited to a single tertiary care center, which may restrict the generalizability of the findings to larger and more diverse populations. The study involved only healthy term newborns, not preterm or low-birth-weight infants, and thus, not all babies who might need early screening were represented. The sample size was also insufficient to establish definitive sensitivity, specificity, positive predictive value, or negative predictive value for early pulse oximetry screening. Because CCHD is relatively uncommon, the present study was not adequately powered to detect rarer CCHD variants or to reliably estimate the risk of false-negative screening results. Also, no long-term follow-up of newborns whose screening was found to be normal was performed, and this could have resulted in missed cases that became clinically apparent after discharge.

Future studies should be multicentric and include larger sample sizes to confirm the applicability of early pulse oximetry screening in different healthcare environments. Such studies should be adequately powered to assess diagnostic accuracy, including sensitivity, specificity, false-positive rates, and false-negative rates for both common and rare forms of CCHD. This should also include high-risk newborn groups, such as preterm babies and low-birth-weight infants. Employing long-term follow-up and cost-effectiveness analyses of early screening protocols would assist in formulating standard protocols to be adopted in universal neonatal care practice.

## Conclusions

The current study shows that early pulse oximetry screening at 15 minutes and six hours of life is a practical method for identifying term newborns with persistent hypoxemia who may require further cardiac evaluation. The results reveal that a two-step screening method is effective in distinguishing newborns with transient low oxygen saturation during the early neonatal transitional period from those with persistent abnormal screening results and minimizes false-positive outcomes related to physiological cardiopulmonary transition after birth. The two-step approach was particularly useful because most newborns with low oxygen saturation at 15 minutes normalized by six hours, supporting repeat assessment before referral for echocardiography. The study confirmed CCHD in 0.37% of the cohort, while additional PDA findings detected during early echocardiography should be interpreted cautiously because follow-up echocardiography was not performed to confirm persistence, spontaneous closure, or need for intervention. Another significant point highlighted by the study is the relevance of repeat evaluation at six hours of life, as it enhances screening interpretation and prevents unwarranted interventions. Pulse oximetry, as an easy-to-use and non-invasive device, can be implemented in routine neonatal care as a screening tool due to its affordability and convenience. These findings should be corroborated by further large-scale studies with adequate follow-up to define diagnostic accuracy, false-negative rates, and the clinical significance of early PDA findings before standard early neonatal screening protocols are recommended for universal practice.
